# Supplementary material for: A Cohort Study on Cancer Incidence among Women Exposed to Environmental Asbestos in Childhood with a Focus on Female Cancers, including Breast Cancer
Source: Int J Environ Res Public Health. 2022 Feb 13;19(4):2086. doi: 10.3390/ijerph19042086 (PMC8872294; doi:10.3390/ijerph19042086)
Supplement: Supplementary file 1 [file ijerph-19-02086-s001.zip › ijerph-1570490-supplementary.pdf]

**Table S1** Cancer incidence among 6024 female former school children compared with a reference group of 54200 females.			
**Cancer site**	**O**	**SIR**	**95% CI**
All cancers (minus other skin)	1331	1.02	0.96 to 1.07
Lip	0	-	-
Tongue	1	0.41	0.06 to 2.90
Mouth	4	0.71	0.26 to 1.88
Salivary glands	2	1.44	0.36 to 5.76
Pharynx	3	0.36	0.12 to 1.12
Oesophagus	3	0.65	0.21 to 2.01
Stomach	12	1.43	0.81 to 2.52
Small intestine	2	0.66	0.16 to 2.64
Colon incl. rectosigmoideum	52	0.97	0.74 to 1.27
Rectum	27	0.94	0.64 to 1.36
Liver	4	0.80	0.30 to 2.12
Gallbladder and biliary tract	5	1.34	0.56 to 3.22
Pancreas	16	0.86	0.52 to 1.40
Anus, other and unspecified digestive organs	5	0.77	0.32 to 1.85
Nasal cavities, middle ear and sinuses	0	-	-
Larynx	4	1.36	0.51 to 3.63
Lung, bronchus and trachea	121	1.11	0.93 to 1.32
Thymus	1	1.45	0.20 to 10.26
Heart and mediastinum	1	1.82	0.26 to 12.93
Bones, joints and articular cartilage	4	1.79	0.67 to 4.78
Melanoma of skin	56	0.87	0.67 to 1.13
Other skin	322	1.08	0.97 to 1.21
Mesothelioma	**6**	**7.26**	**3.26 to 16.15**
Peripheral nerves and autonomic nervous system	0	-	-
Peritoneum and retroperitoneum	4	2.01	0.75 to 5.34
Other connective tissue	7	1.29	0.62 to 2.71
Breast	343	0.98	0.89 to 1.09
External female genital organs and vagina	11	1.71	0.94 to 3.08
Cervix uteri	50	1.11	0.84 to 1.46
Corpus uteri	**61**	**1.29**	**1.01 to 1.66**
Ovary, fallopian tube and broad ligament	34	0.72	0.52 to 1.01
Other and unspecified female genital organs	1	4.39	0.62 to 31.14
Kidney	12	1.05	0.59 to 1.84
Renal pelvis and ureter	2	0.79	0.20 to 3.17
Urinary bladder	21	1.12	0.73 to 1.71
Other and unspecified urinary organs	**2**	**4.27**	**1.07 to 17.07**
Eye	0	-	-
Meninges	12	0.64	0.36 to 1.12
Brain	20	0.86	0.56 to 1.33

Other parts of CNS	5	0.73	0.31 to 1.77
Thyroid gland	17	1.46	0.91 to 2.35
Adrenal gland	1	1.68	0.24 to 11.90
Hodgkin's lymphoma	7	1.55	0.74 to 3.25
Non-Hodgkin lymphoma and malignant immunoproliferative disease	20	0.93	0.60 to 1.45
Multiple myeloma	**2**	**0.24**	**0.06 to 0.95**
Lymphatic leukaemia	6	0.69	0.31 to 1.53
Myeloid leukaemia	12	1.72	0.98 to 3.02
Monocytic leukaemia	1	2.11	0.30 to 14.96
Other and unspecified leukaemia	1	4.26	0.60 to 30.25
Other and unspecified cancer in lymphatic and haematopoietic tissue	2	-	-
Ill-defined and unspecified cancer	26	1.03	0.70 to 1.51

- Indicates that there are no observations in one of the cohorts.

Abbreviations: CI, confidence interval; O, observed number of cases; SIR, standardized incidence ratio

Bold denotes statistically significant results.
